# Evaluation of Pain Mitigation Strategies in Goat Kids after Cautery Disbudding

**DOI:** 10.3390/ani10020277

**Published:** 2020-02-11

**Authors:** Inês Ajuda, Monica Battini, Silvana Mattiello, Cecilia Arcuri, George Stilwell

**Affiliations:** 1 Animal Behaviour and Welfare Laboratory, Centre of Interdisciplinary Research in Animal Health, Faculty of Veterinary Medicine, Lisbon University, 1300-477 Lisbon, Portugal; stilwell@fmv.ulisboa.pt; 2 Università degli Studi di Milano, Department of Agricultural and Environmental Sciences - Production, Landscape, Agroenergy, Via G. Celoria 2, 20133 Milan, Italy; monica.battini@unimi.it (M.B.); silvana.mattiello@unimi.it (S.M.); arcuri.cecilia@yahoo.it (C.A.)

**Keywords:** disbudding, pain mitigation, analgesia, anaesthesia, goat kids

## Abstract

**Simple Summary:**

Disbudding is a routine procedure performed in goat kids at an early age, especially the ones in the dairy industry. The procedure is mainly done to increase safety for other animals and workers in intensive dairy farms. Disbudding is a painful procedure that affects the welfare of the kids. Effective and practical pain mitigation strategies to reduce the suffering of goat kids due to disbudding have not yet been found. We studied two different pain mitigation strategies for this procedure and concluded that they were not entirely effective. Consumers are increasingly aware of animal farming practices, especially the ones that can lead to suffering and pain, such as disbudding. It is crucial that pain mitigation strategies as well as possible alternative solutions to disbudding continue to be investigated.

**Abstract:**

Nowadays, most of the goat milk production in developed countries is done in intensive indoors production systems. In these systems, procedures such as disbudding are performed routinely. Disbudding is done in young goat kids and is a recognised as a painful procedure. Pain mitigation strategies have been extensively researched, but a method that is effective in mitigating pain as well as being safe and practical has not yet been found. In this paper we used three treatment groups: one control and two groups with pain mitigation strategies for cautery disbudding, one using local anaesthesia (lidocaine) and a second one using local anaesthesia (lidocaine) plus an analgesic (flunixin meglumine). The behaviour of twenty-seven goat kids was recorded for three hours after disbudding. Overall, the goat kids that received both pain mitigation treatments dedicated more time performing active and positive behaviours. Nevertheless, the incidence of behaviours related to pain and discomfort was not consistently reduced. Research is still needed to find a practical and effective pain mitigation strategy for disbudding. A solution to this challenge would improve animal welfare as well as address societal concerns linked to the suffering of farm animals.

## 1. Introduction 

Disbudding, the process of burning the horn buds, is a routine procedure performed in dairy goat farms for many reasons, including reducing the risk of injuries to other animals (e.g., bruises), to the animal itself (e.g., getting caught in fences), as well as to stockpeople [1], and the increased need for space during resting and feeding for horned animals [2,3,4]. 

In spite of these advantages, disbudding has been recognised as a painful procedure [5]. However, although farmers have the responsibility of minimising the pain of their animals [6], this procedure is frequently carried out without effective pain mitigation [2,7]. According to the International Association for the Study of Pain (IASP), “pain is an unpleasant sensory and emotional experience associated with actual or potential tissue damage” [8] and can have a great impact on the animal’s quality of life [9]. In some cattle breeds, hornless animals can be bred, avoiding the need for disbudding. Unfortunately, this cannot be achieved in goats, as the genes associated with hornless (polled) animals are also associated with a recessive gene for intersex [10]. Furthermore, disbudding has proven to be more challenging for goat kids than for calves, due to a less mature skull of the kids at the time of disbudding [11], to the position of the horns (a more parietal position) and to the fact that horn buds in goat kids are proportionally larger than in calves [12,13]. Therefore, disbudding goat kids with a hot cautery iron (to destroy the horn bud cells) has a higher risk of causing severe burns in the brain tissue [7] that may lead to acute as well as chronic pain [5]. For these reasons, the use of analgesia and anaesthesia is always recommended when disbudding goat kids [14] and the Council of Europe [15] states that “unless the existing national legal system allows otherwise, disbudding and castration shall only be carried out by a veterinarian using an anaesthetic” and that “if disbudding is to be carried out it should be done as soon as the bud is sufficiently developed for the operation to be effective”. However, according to the European legislation, “due to the anatomy of the kids’ skull, disbudding even under anaesthesia is a difficult procedure” [15]. 

Pain mitigation in the disbudding of dairy calves has proven to be highly effective [16,17,18], but when it comes to disbudding goat kids, this has not always been successful [2,7,19,20]. Several methodologies (meloxicam [20], lignocaine [19,21], clove oil essence [22]) have been assessed through physiological and behavioural indicators and a mixture of xylazine and ketamine [2] has been tested in different purpose breeds (French alpine and Saanen [19], Beetal [21] and Swedish Landrace [20]), demonstrating conflicting results ranging from short-lasting effect of the pain mitigation [20] to better performance [21]. Finally, isoflurane has proven to be very effective, either alone or in combination with meloxicam [23]. Although this method can be quite effective, it can present some challenges in cost, the applicability on farms and the need of vet assistance.

Frequently used measures of pain in animals include behavioural and physiological measures [7]. Cortisol assessment is commonly used to assess stress associated with pain that stimulates the hypothalamic pituitary adrenal axis [24], but it can also have its limitations, such as responding to other type of stimuli like the reproductive cycle, handling and restraining [25], as well as circadian changes. Behaviour indicators are less invasive and can be more specific to different types of pain [26] than physiological indicators, offering a useful means of pain assessment in farm animals [27]. Behavioural indicators have been validated for the disbudding of dairy calves (reviewed by Stafford and Mellor, 2011) and dairy goat kids [7].

Effective pain mitigation with widely available drugs that can be easily administered by trained veterinarians can impact tremendously on dairy goat kid welfare worldwide. We hypothesised that disbudding goat kids in a commercial dairy system with pain management provided by local anaesthesia (lidocaine) alone or combined with an analgesic (intravenous flunixin meglumine) could mitigate the acute pain from the hot iron disbudding up to three hours after the disbudding procedure.

## 2. Materials and Methods 

### 2.1. Location, Farm and Animals 

The study was conducted at a commercial dairy goat farm located Benavente (38.9817° N, 8.8096° W), in the south of Portugal. The animal welfare and ethics committee of the Faculdade de Medicina Veterinária (Universidade de Lisboa) approved the experimental protocol (approval no. 266). A total of 27 goat kids (8–14 days of age; French Alpine (*n* = 15) and Saanen (*n* = 12); males (*n* = 13) and females (*n* = 14)) were included. All kids were separated from their mother at birth and fed by an artificial feeding system (milk ad libitum). During the study, the kids were housed in 16 m^2^ pens (always in a group, maximum 15 kids/pen) with water and an automatic milk dispenser, both available ad libitum, and slatted plastic floors. The kids were randomly allocated to the 3 treatments, placed in 2 pens throughout the experiment and marked with numbers from 1 to 15 in each pen. They were disbudded in the pens while restrained. The experiment was carried out on the same day.

### 2.2. Treatment Protocols

Goat kids were randomly allocated to one of the three treatment protocols (*n* = 9 goat kids per treatment), balancing breed and sex. Animals did not undergo a previous handling or sampling habituation plan. Treatments consisted of: Group Control (GC)—1 mL of saline solution injected over the cornual branches of the lacrimal and infratrochlear nerves (zygomaticotemporal - lacrimal and infratrochlear) of each horn (2 mL/horn in total) 15 min before the disbudding procedure by thermal cauterization. One injection was applied midway between the lateral canthus of the eye and the lateral base of the horn bud. The second injection was applied at the frontal base of the horn bud, at approximately the medial canthus [19]. Group Lidocaine (GL)—Group Lidocaine (GL)-injections were made at the same sites and with the same volume as the Control group, but lidocaine (Anestesin 2%) was injected instead of saline solution. Group Lidocaine_Flunixin (GL+F) followed the same protocol as for GL but, additionally, an intramuscular injection of flunixin meglumine (0.08 mL; Meflosyl 5%, 50 mg/mL, Zoetis^®^, Lisbon, Portugal), was given. During injections and disbudding, the kid was gently held and restrained by a trained vet. Disbudding was done using an electrically heated dehorner (Goat Dehorner, Lenk^®^ 200 GD) that was applied two times (8–10 s each time) per bud, and the area was allowed to cool down for at least 5 s before re-application. The disbudding was considered sufficient when the corium of the bud was completely cauterized and removed [28]. After disbudding, each wound was sprayed with a topical antibiotic (Oxytetracycline hydrochloride, 3.92%, Terramycin^®^ Aerosol spray). 

### 2.3. Behavioural Recording and Monitoring

All kids were filmed using cameras (SONY^®^ HANDYCAM HDR-PJ410) fixed to a corner of each pen, for three hours after the end of the disbudding, in order to record pain-related behavioural events, as well as any positive engagement. The videos were analysed by a trained assessor who was not aware of the treatments used, using the free BORIS software (Behavioural Observation Research Interactive Software [29]). Initially it was essential to define an ethogram, associating each behaviour with a key on the keyboard. The ethogram is composed of state events (quantified in duration, expressed in seconds) and point events (quantified as absolute frequencies). Some behaviours have been considered mutually exclusive, as summarized in Table 1.

### 2.4. Statistical Analysis 

State behaviours were expressed in terms of percentage of time, number of bouts and average duration of each bout, while point events were expressed as frequency of occurrence. The ethological data collected were exported to Excel and tabulated to allow further analysis.

A kid belonging to GL was eliminated from the analysis, having not been visible for most of the observation time. Furthermore, before proceeding to the statistical processing, some behaviours were eliminated, as they never occurred (Self-grooming), or were merged with others, as their frequency would have been too low to be processed individually. Social Play was merged with Play in the “Play” category, and Non-alert lying was considered as a single behaviour, regardless of the position of the head (up or down).

In order to compare the effect of the treatments, state behaviours were analysed by one-way ANOVA, while the event behaviours were analysed by non-parametric variance (Kruskal–Wallis test), and multiple comparisons were performed using LSD test. Furthermore, the average duration of state behaviours was also analysed by Principal Component Analysis (PCA).

## 3. Results

**Figure 1:**
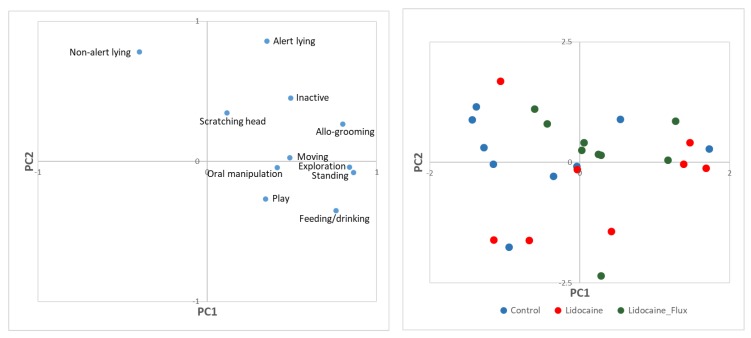
Results of principal component analysis (PCA) (loading plot, on the left; score plot, on the right) performed on the percentages of the time dedicated to state behaviours in the three treatments during the observation period.

Disbudding could be effectively achieved without problems in all kids. Figure 1 shows the PCA results relating to the percentage of time dedicated to state behaviours. 

The descriptive statistics for state behaviours relating to the percentage of the time, the average number of bouts and average duration of each bout in the three treatments are reported in Table 2, Table 3 and Table 4, respectively. 

GC kids showed a significantly lower percentage of time spent standing compared to both treated groups, while no significant differences were observed between the two treated groups (Table 2). This agrees with the higher number of standing bouts in treated kids, with significant differences between GC vs GL, differences approaching statistical significance between GC and GL+F, and no differences between the two treated groups (Table 3). No difference was recorded as to the mean duration of each bout (Table 4).

Treated kids scratched their heads more often than those of CG, with significant differences between GC and GL+F (*p* = 0.035), but only limited differences between GC and GL+F (*p* = 0.095) (Table 2). No differences were recorded in the number of bouts (Table 3), nor in their mean duration (Table 4). The percentage of time spent playing and the number of bouts is higher in treated kids, with significant differences between GC and GL+F (*p* = 0.043 and 0.015, respectively), while the average duration of bouts does not differ between treatments (Table 2, Table 3 and Table 4). Feeding/drinking is higher in the goat kids that received lidocaine and lidocaine plus flunixin meglumine (GL and GL+F). Although the differences in the total percentage of time are not statistically significant, a significantly higher number of bouts is observed in GL compared to GC, while the differences between GL+F and GC were did not statistical significance (*p* = 0.097). 

No significant differences between groups were observed for point events (Table 5). The abnormally high frequency of vocalisations in GL was due to a single individual that emitted 146 vocalisations during the three-hour observation period, for reasons that we were not able to identify.

## 4. Discussion

In spite of the difficulties reported in literature for disbudding goat kids due to the anatomy of the kids’ skull [11,12], the procedures followed in the present study were effective [7] and no complications were observed. Furthermore, the double injection of anaesthetic drugs (at the cornual branches of the lacrimal and infratrochlear nerves) and the drug and doses adopted for this study were effective to mitigate pain in treated kids after being disbudded, in spite of the difficulties reported by Matthews & Duncan (2019) [13], deriving from the peculiar distribution of nerves to the horns.

In general, the PCA on the percentages of time dedicated to the various behaviours shows a tendency for the control group to scatter on the left side of PC1, characterized by higher values of non-alert lying and lower values of more active behaviours, in particular of standing, exploring, feeding/drinking, playing and allo-grooming (Figure 1 and Table 1 and Table 2). 

Inactivity is widely recognised as a sign of pain in animals [30,31,32]. In the present study, the kids that received drugs for pain management spent more time standing than the kids that did not. This is in agreement with the trend of treated kids to spend less time in non-alert lying, although this difference was not statistically significant, probably due to the high individual variation (Table 1). In dairy calves, Morisse and colleagues (1995) [33] found no difference in the ratio of standing to lying between two 24-hour periods of observation (before and after disbudding), but treated calves had a higher ratio of standing/lying, showing a longer time spent standing. Chandrahas et al. [34] found that kids that received lidocaine and meloxicam had a similar standing time than the control kids in the first hours after disbudding, with the group treatment that had the higher standing time varying from hour to hour, on the first three hours of observations and also at the fourth hour of observation. The authors suggested these behavioural responses were due to increased restlessness or reduced comfort related to the procedure, as well as the different timings of actions of the drugs (starting time or time when the effect of the drug starts to wear off). McMeekan et al. [35] also reported that disbudded calves spent more time lying than handled controls for up to 4 hours post-treatment. Still in dairy calves, Stilwell et al. [36] found that non-treated caustic-paste disbudded calves showed an “inert-lying” posture that was not evident in those that received analgesia. 

In goats, Hempstead et al. [7] found a tendency, although not significant, for kids that had been disbudded with a hot iron to lay down more than kids that had been sham disbudded, hypothesising that the kids were conserving energy for repair of damage caused by disbudding or the kids were keeping their sensitive heads from moving thereby exacerbating the pain, which seems to be the case for this study as well. Nevertheless, for other painful mutilations performed in other production systems, such as castration of piglets and lambs, lying time was higher than for handled controls that did not receive any painful procedure [24,37]. 

In addition to indicators that may be indicative of a state of pain, other welfare indicators are relevant and important to measure. For example, positive welfare indicators should be included in welfare evaluations, as they can help to distinguish between a situation that is solely a result of the absence of negative experiences and a situation where positive experiences or sensations are present [33]. In our study, positive behaviour indicators (exploration, feeding/drinking and play) were higher in goat kids that received pain management. Exploration (climbing up structures, sniffing or licking the housing structures) was particularly high in GL kids, as the lack of analgesic treatment in addition to the local anaesthetic treatment did not prevent them from exploring and interacting with the environment. In agreement with our findings, Mintline and colleagues [38] found a higher exploration rate in disbudded dairy calves that received anaesthesia and analgesia, interpreted by the authors as an indicator of pain mitigation. Chandrahas et al. [34] also found similar results with goat kids that only received lidocaine having a mean exploration time significantly higher than any other treatment (control and other types of pain mitigation, such as analgesia with meloxicam). 

Other positive behaviours, i.e., play behaviour and feeding, were also significantly higher in kids that received pain mitigation. Consistent with our results, a reduction in feeding time after disbudding was recorded in dairy calves [16] and in goat kids [7,11], in groups of animals that were disbudded without a pain mitigation treatment or a pain mitigation treatment that was less effective. As to play behaviour, researchers [33] report that this activity is absent in humans and non-human mammals when events that threaten their health and fitness are present. Mintline et al. [38] found a reduction in play behaviour in dairy calves after disbudding, independent of the level of activity. Therefore, the higher proportion of time dedicated to this behaviour by GL and GL+F suggests that both treatments had a positive effect on pain management. 

The frequency and the average time spent head scratching were higher in kids that received either anaesthesia (with significant differences from the control group) or anaesthesia and analgesia. The frequency of head-directed behaviours, including head scratching, was considered by some [7,20] as a good indicator of pain linked to disbudding. However, in a more recent paper [11], the same authors have concluded that, when comparing different kinds of pain management, no pain management and sham disbudding, head scratching could not be considered as a clear pain indicator. Our results seem to confirm that an increase in this behaviour is not necessarily related to an increase of pain or discomfort. On the contrary, the high frequency of scratching may indicate a level of discomfort that is representative of an attenuated level of pain. Scratching has been described as a comfort and maintenance behaviour in goats and other ruminants [33,39,40]. It has also been associated with diseases that cause mild pain or discomfort at skin level [41]. In this study the authors hypothesise that the higher level of head scratching in treated kids was due to a higher toleration to touch on the burned area due to the pain mitigation action. At the same time, the fact that attention is drawn to that area of the head may suggest the goat kids were still feeling some kind of discomfort or even pain. 

Finally, the lower frequency of body shaking in control kids is apparently in contrast with the findings by Hempstead et al. (2017) [7], who reported a reduction in body shaking following cautery disbudding, as the animal’s attention is being diverted to the head region rather than to maintenance behaviours such as body shaking. In our results, the low frequency of this behaviour can probably be related to the general low level of activity of the not-treated kids, as discussed above.

In this instance, data collected during disbudding was not collected due to the setup of the trial. During disbudding, goat kids were constrained in a non-transparent constraining device, which would alter any results that could be recorded such as behaviour observation or sound recording. 

## 5. Conclusions

Overall, the goat kids that received both pain mitigation treatments dedicated more time performing active (standing) and positive (exploring, playing, feeding) behaviours. Nevertheless, the presence of behaviours such as head scratching (although the higher frequency may demonstrate a higher tolerance to touch in the burnt area) still demonstrates some level of discomfort and possibly pain. In agreement with previous findings, the present research confirms that, to a certain extent, providing pain mitigation can improve the animal welfare status of the goat kids after disbudding. Further research is needed to identify safe and effective pain mitigation methods for disbudding goat kids. These alternatives will improve the welfare of the goat kids as well as address societal concerns regarding the suffering of animals in agriculture. 

## Figures and Tables

**Table 1:** List of behaviours (state or point events) monitored during the experiment and their definitions and codes. Mutually exclusive behaviours are reported in the column “Excluded behaviours”.

Behaviour Code	Behaviour Type	Description	Key	Excluded Behaviours
Alert lying	State event	The kid is lying down, eyes open, head up, reactive to external stimuli	V	A, D, G, P, M, 0, S
Allo-grooming	State event	The kid is licking or sniffing other kids	L	A, D, E, G, P, T, I, O, 0, F
Evacuation	Point event	The kid urinates or defecates	U	
Exploration	State event	The kid is licking or sniffing the housing structures or is climbing up	E	A, L, D, G, P, T, I, O, 0, F
Feeding/drinking	State event	The kid’s head is at the feed rack or at the drinker	A	L, D, V, E, G, P, T, I, O, M, 0, F
Inactive	State event	The kid is idling inactive and does not interact with the environment nor with other kids	I	A, L, D, V, E, G, P, T, O, M, 0, F
Moving	State event	The kid is walking or running to move around	M	A, L, D, V, G, P, T, I, O, 0, F
Moving the tail	Point event	The kid is moving the tail	C	
Non-alert lying	State event	The kid is lying down, eyes closed, nonreactive to any external stimulus. 1 = head up; 2 = head down (on the soil or on its own body)	D	A, L, V, E, G, P T, I O, M, 0, S, F
Nonvisible	State event	The kid is not visible (zero), e.g., hidden in a blind corner	0	A, L, D, V, E, G, P, T, I, O, M, F, S
Oral manipulation	State event	The kid is biting or chewing an object or the litter, or is moving its mouth with no apparent purpose	O	A, L, D, V, E, G, P, T, I, M, 0, F
Play	State event	The kid is playing alone (runs, jumps on the walls or in the air)	P	A, L, D, V, E, G, T, I, O, M, 0, F
Scratching head	State event	The kid is scratching its head with the legs or against an object	T	A, L, D, V, E, G, P, I, O, M, 0, F
Self-grooming	State event	The kid is licking itself (any part of the body)	F	A, L, D, V, E, G, P, T, I, O, M
Shaking	Point event	The kid shakes the head or ears	Q	
Social play	State event	The kid is playing with other kids or is encouraging them to play (runs, chases, jumps, play flights)	G	F
Standing	State event	The kid is standing on four legs	S	D, V, 0
Stargazing	Point event	The kid brings the head back, looks up, with no visual stimulus present	X	
Stretching	Point event	The kid is stretching	Z	
Vocalisation	Point event	The kid emits any type of sound	B	
Yawning	Point event	The kid yawns	Y	

**Table 2:** Average percentage (± SD) of the time of manifestation of each state behaviour in the three treatments during the observation period, and relative levels of significance of the differences. Significance: * *p* < 0.05; letters (a and b) on the same line indicate statistically significant differences (LSD test).

Behaviour	Control-GC (*n* = 9)	Lidocaine-GL (*n* = 8)	Lidocaine_Flux GL+F (*n* = 9)	*p* Value
Feeding/drinking	4.39 ± 3.41	5.85 ± 3.75	5.63 ± 2.32	0.594
Allo-grooming	1.12 ± 1.25	1.51 ± 1.23	1.29 ± 0.72	0.764
Non-alert lying	43.49 ± 29.48	29.05 ± 28.15	34.03 ± 17.36	0.501
Alert lying	24.46 ± 14.98	21.29 ± 16.55	29.32 ± 11.69	0.521
Exploration	2.66 ± 1.95^a^	6.25 ± 4.57^b^	4.92 ± 3.04^ab^	0.096
Playing	0.52 ± 0.81^a^	1.50 ± 1.81^ab^	1.94 ± 1.46^b^	0.113
Scratching head	0.68 ± 0.71^a^	1.74 ± 1.82^ab^	3.04 ± 3.18^b^	0.089
Inactive	17.32 ± 13.04	13.01 ± 11.84	20.72 ± 13.59	0.480
Oral manipulation	0.77 ± 1.03	0.44 ± 1.04	0.60 ± 0.81	0.783
Moving	2.52 ± 2.78	2.85 ± 1.40	2.78 ± 1.33	0.936
Standing	10.58 ± 5.48^a^	19.05 ± 7.47^b^	18.21 ± 5.25^b^	0.014*

**Table 3:** Average number (± SD) of bouts of each state behaviour in the three treatments during the observation period, and relative significance levels of the differences. Significance: * *p* < 0.05; letters (a and b) on the same line indicate statistically significant differences (LSD test).

Behaviour	Control-GC (*n* = 9)	Lidocaine-GL (*n* = 8)	Lidocaine_Flux GL+F (*n* = 9)	*p* Value
Feeding/drinking	8.22 ± 5.61^a^	15.38 ± 8.67^b^	13.67 ± 5.59^ab^	0.090
Allo-grooming	10.11 ± 8.04	16.25 ±11.55	14.11 ± 6.62	0.361
Non-alert lying	8.33 ± 4.50	8.13 ± 8.24	8.56 ± 4.45	0.989
Alert lying	19.11 ± 12.49	21.38 ± 17.84	18.44 ± 7.60	0.890
Exploration	18.44 ± 10.63^a^	39.88 ± 21.25^b^	31.11 ± 13.02^ab^	0.030*
Playing	4.44 ± 4.28^a^	12.00 ± 11.20^ab^	13.56 ± 8.13^b^	0.062
Scratching head	11.89 ± 11.43	30.50 ± 33.37	33.22 ± 28.87	0.192
Inactive	25.11 ± 15.69	27.00 ± 18.68	28.33 ± 17.39	0.924
Oral manipulation	1.78 ± 2.77	1.75 ± 2.49	2.78 ± 3.73	0.729
Moving	31.11 ± 18.72^a^	65.50 ± 38.95^b^	56.44 ± 33.81^ab^	0.081
Standing	39.44 ± 19.91^a^	76.38 ± 38.50^b^	63.89 ± 28.23^b^	0.047*

**Table 4:** Average duration (± SD), expressed in seconds, of each bout of manifestation of state behaviour in the three treatments during the observation period, and relative levels of significance of the differences. Significance: * *p* < 0.05; letters (a and b) on the same line indicate statistically significant differences.

Behaviour	Control-GC (*n* = 9)	Lidocaine-GL (*n* = 8)	Lidocaine_Flux-GL+F (*n* = 9)	*p* Value
Feeding/drinking	49.45 ± 33.23	34.14 ± 19.95	37.99 ± 14.25	0.400
Allo-grooming	8.18 ± 4.62	8.34 ± 3.03	8.07 ± 2.18	0.987
Non-alert lying	411.89 ± 242.49	346.28 ± 348.39	360.04 ± 181.40	0.860
Alert lying	134.29 ± 120.13	77.35 ± 52.86	142.53 ± 62.82	0 .256
Exploration	12.69 ± 4.92	13.18 ± 6.07	14.02 ± 7.65	0.904
Playing	0.08 ± 0.07	0.11 ± 0.07	0.16 ± 0.13	0.238
Scratching head	5.43 ± 3.13	5.03 ± 1.09	7.09 ± 3.36	0.283
Inactive	59.36 ± 52.29	34.31 ± 20.38	61.08 ± 46.17	0.374
Oral manipulation	27.62 ± 51.56	8.96 ± 12.96	10.50 ± 11.81	0.413
Moving	6.86 ± 3.25^a^	4.12 ± 1.40^b^	4.66 ± 1.01^b^	0.033*
Standing	26.40 ± 10.19	23.34 ± 6.99	27.91 ± 8.78	0.565

**Table 5:** Average number (± SD) of manifestation of each event for each group during the observation period and respective *p* value.

Behaviour.	Control-GC (*n* = 9)	Lidocaine-GL (*n* = 8)	Lidocaine_Flux (GL+F) (*n* = 9)	*p* Value
Eliminatory Behaviour	1.33 ± 1.22	1.75 ± 0.71	1.78 ± 1.48	0.705
Moving tail	39.00 ± 44.59	36.63 ± 31.00	47.78 ± 33.93	0.720
Yawning	0.00 ± 0.00	0.25 ± 0.71	0.22 ± 0.67	0.573
Shaking	14.33 ± 12.92	25.00 ± 24.91	29.22 ± 11.78	0.133
Stargazing	0.78 ± 1.30	0.63 ± 0.916	1.67 ± 2.83	0.980
Stretching	1.44 ± 1.42	1.38 ± 1.19	1.44 ± 1.88	0.936
Vocalization	0.33 ± 0.50	19.25 ± 51.22	0.67 ± 1.00	0.105
